# A Spiritual care department: practical experience

**DOI:** 10.1136/spcare-2024-004809

**Published:** 2024-03-01

**Authors:** Nadereh Memaryan, Fatemeh Rezaei Khodadadi

**Affiliations:** 1 Spiritual Health Research Center, Mental Health Department, School of Behavioral Sciences and Mental Health (Tehran Institute of Psychiatry), Iran University of Medical Sciences, Tehran, Iran (the Islamic Republic of); 2 Spiritual Health Research Center, Hemmat Highway next to Milad Tower, Iran University of Medical Sciences, Tehran, Iran (the Islamic Republic of)

Spiritual care identifies and responds to humans’ spiritual needs, including the need for communication, emotional support, respect for values and beliefs, and search for meaning in life.^1^ This type of care may consist of respecting the patients’ beliefs, communicating effectively by reflective listening and talking to the patients, accompanying them through compassionate care, supporting them, showing empathy, facilitating their participation in religious rituals, promoting their sense of good and usefulness, and if need be, referring to clergies and other experts.^2^ There is convincing evidence that many patients and their families have spiritual needs that will help with their effective recovery if properly considered in healthcare services.^3^ Patients need to adapt to their illness, receive compassion, find meaning in their life, and learn the answers to their numerous questions, such as why they have developed this illness.

In recent decades, spiritual health and spiritual care have received more attention in healthcare services.^4^ Therefore, to create meaning in the patients’ lives and due to beneficial effects on their health, hospitals, which are a key part of the healthcare system, must identify the patients’ spiritual needs and move towards the delivery of spiritual care to them.^5^

The delivery of spiritual care is a subject that has received considerable attention in Iran in recent years. Although many scientific documents have been published on this issue,^6^ there was no spiritual care department in the Iranian healthcare system to provide these services in line with the patients’ spiritual needs, backed up by research, and based on the existing conditions and cultural and religious considerations. This deficiency was further corroborated by our review of the experiences surrounding the provision of spiritual care at the Departments of Spiritual Care of the National Institutes of Health (NIH) Clinical Centre, Johns Hopkins Hospital, and the University of Michigan in the USA and also the Department of Spiritual Care of Vancouver General Hospital in Canada.

At the Spiritual Health Research Centre of Iran University of Medical Sciences, we first designed a spiritual care guideline for cancer patients,^7^ which included the spiritual assessment of patients and defining key interventions in spiritual care. In another study, we explained the implementation and integration of this programme in hospital departments by presenting a flowchart of the service and the processes before, during, and after the action. After the successful experience of administering spiritual care during the COVID-19 pandemic at Rasoul Akram Hospital in Tehran (one of the main hospitals affiliated with this university and among the largest teaching hospitals in Iran), the provision of spiritual care services began on a permanent basis, and not just as a research project. The activities of the Spiritual Care Department focused on planning for service provision and referrals to receive these services, defining the characteristics of the workforce providing this service in a precise manner, the selection of this workforce and offering the necessary training to them, evaluating the effectiveness of the service and developing it in different hospital departments, and group supervision meetings for monitoring purposes.

Human resources are the principal component of every service-delivery unit. In the described experience, the service provider and support team is multidisciplinary, consisting of social medicine experts, master’s degree and PhD holders in health and clinical psychology, PhD holders in theology, psychiatry, philosophy and *kalam* (Islamic scholastic theology) and members of the clergy. First-line spiritual caregivers include psychologists trained in spirituality; second-line spiritual caregivers, who deal with referrals from the first line, are members of the clergy trained in health.

Documenting, presenting and reviewing reports are essential for any public service provision and its evaluation. As such, a special spiritual care system was designed to record the necessary information, including the patients’ key details (using codes to ensure their confidentiality) as well as the service provider’s information, a description of the spiritual history and care service received, referrals, and special considerations for each patient. Evaluation experts can monitor this information online in the system.

Services for everyone, constant learning, and striving for better outcomes are the values underlying this department, which are regularly reminded and reviewed by the members in their weekly group supervision meetings.

## References

[1] Kuepfer J , Schmidt A , O’Connor TSJ , et al . Promise, provision, and potential: a hopeful trajectory for spiritual care in long-term care. J Pastoral Care Counsel 2022;76:105–13. 10.1177/15423050221090870 35379026 PMC9158234

[2] Attard J , Baldacchino DR , Camilleri L . Nurses' and midwives' acquisition of competency in spiritual care: a focus on education. Nurse Educ Today 2014;34:1460–6. 10.1016/j.nedt.2014.04.015 24814103

[3] Timmins F , Connolly M , Palmisano S , et al . Providing spiritual care to in-hospital patients during COVID-19: a preliminary European fact-finding study. J Relig Health 2022;61:2212–32. 10.1007/s10943-022-01553-1 35511386 PMC9069948

[4] Fitch MI , Bartlett R . Patient perspectives about spirituality and spiritual care. Asia Pac J Oncol Nurs 2019;6:111–21. 10.4103/apjon.apjon_62_18 30931354 PMC6371668

[5] Adib-Hajbaghery M , Zehtabchi S . Assessment of nurses’ professional competence in spiritual care in Kashan’s hospitals in 2014. Avicenna J Nursing Midwifery Care 2014;22:23–32. 10.1177/0969733015600910

[6] Moeini M , Taleghani F , Mehrabi T , et al . Effect of a spiritual care program on levels of anxiety in patients with leukemia. Iran J Nurs Midwifery Res 2014;19:88–93. 24554966 PMC3917191

[7] Memaryan N , Jolfaei AG , Ghaempanah Z , et al . Spiritual care for cancer patients in Iran. Asian Pac J Cancer Prev 2016;17:4289–94. 27797232

